# Smithsonian Microbeam Standards

**DOI:** 10.6028/jres.107.054

**Published:** 2002-12-01

**Authors:** Eugene Jarosewich

**Affiliations:** Department of Mineral Sciences, NMNH, Smithsonian Institution, Washington, DC 20560

**Keywords:** electron microprobe standards, microbeam standards, mineral standards, reference materials

## Abstract

This is a short history of the Smithsonian Microbeam Standards; their sources, selection, preparation, and analyses. Fifty-eight minerals, natural glasses, and synthetic samples have been characterized in the past 25 years. During that time, over 750 requests were received for approximately 11 000 individual samples. These reference samples are referred to as the Smithsonian Microbeam Standards.

## 1. Introduction

The early 1960s was a period when various Federal agencies generously supported the development of new instrumentation and new techniques for the analysis of lunar samples whose return was anticipated. In the fall of 1964, the Department of Mineral Sciences, National Museum of Natural History, with funding from NASA, purchased an Applied Research Laboratory electron microprobe and expanded its laboratory facilities for the study of meteorites. By then the electron microprobe had become an established instrument and most of the basic analytical techniques were already developed. Since x-ray microanalysis is not based on first principles of physics or chemistry, but relies on comparison with materials of known composition, a set of well-characterized standards is required for analysis of unknowns. At this early stage of electron microprobe analyses only a few mineral standards were available. In the following years, the National Mineral Collection at the Smithsonian served as an invaluable source of minerals for reference samples. Although the laboratory staff focused primarily on meteorite research, they also analyzed minerals, mineral separates, and natural glasses, several of which became electron-microprobe standards. These analyzed minerals and natural glasses were initially intended to serve our own needs; however, numerous requests for these standards prompted the staff to publish the data and make small quantities available to interested researchers.

As a rule, materials selected for standards should be analyzed by more than one laboratory and, if possible, by two independent methods. However, in our setting this ideal approach was constrained by both insufficient funds and the limited amount of many samples. For these reasons most samples were analyzed only once by wet-chemical methods. Based on our experience, a careful wet-chemical analysis will provide satisfactory major-element results. The only samples in our suite of standards analyzed by more than one laboratory or different analysts were: Cr-bearing augite from Nevada; A-99 basaltic glass from Hawaii; and partial analyses of hornblende, pyrope, and augite from Kakanui, New Zealand, and VG-2 glass from the Juan de Fuca Ridge.

## 2. Standards

As the staff’s interest in electron-microprobe analyses of different geological materials expanded, the demand for additional standards increased. For example, in the late 1960s George Switzer, then chairman of our department, obtained questionable results with the available standards when analyzing garnets from diamond-bearing kimberlites in South Africa. He addressed this problem by preparing mineral separates of two garnets and an omphacite, since no suitable garnet and omphacite specimens were available. These three separates were then analyzed by wet-chemical methods and thereafter used as standards. When using these “like standards”, of close composition to the unknowns, acceptable results were obtained as judged by stochiometry and analytical totals close to 100 %.

About the same time Brian Mason, then curator of the National Collection of Meteorites, considered re-analysis of hornblende and pyrope from Kakanui, New Zealand. He felt that the values for these two minerals in his original publication [1] were in error. After reanalysis, these two minerals plus the garnets and omphacite were routinely used as standards in subsequent studies [2].

Further work in developing accurate standards were undertaken in the early 1970s, as Bill Melson, curator of the Petrology collection, began a major study of seafloor volcanic glasses. One of the objectives of this work was to determine compositions of glasses from different localities around the globe. Basaltic glasses VG-2 and A-99 were selected as standards for this project [3]. Several institutions used VG-2 as a standard for electron-microprobe analyses, and in order to assure their quality, round-robin analyses were undertaken by three laboratories. A polished disk with VG-2, A-99, Kakanui hornblende, and two glasses of unknown composition was analyzed by the United States Geological Survey (Reston, VA), the Massachusetts Institute of Technology, and the Smithsonian Institution. In order to determine the precision and accuracy of analyses, the samples were first analyzed with Kakanui hornblende as the standard for precision (uncorrected results), and then with the preferred standards of each laboratory and finally compared with wet-chemical analyses (Smithsonian). The round-robin test revealed excellent agreement in the precision of analyses from the three laboratories when the samples were analyzed with Kakanui hornblende as the standard. When the samples were analyzed with each laboratory’s own standards, some with widely different compositions from those of the unknowns, there were considerable matrix corrections. Nevertheless, the results among the three laboratories for the two unknowns and the three standards were in good agreement with the wet-chemical analyses [4].

As geochemical research at the Smithsonian broadened, it became clear that a wide range of well characterized materials were needed as primary and secondary standards for electron-microprobe analyses, as well as standards for special applications. Also, since a large number of requests were received for different standards, J. Nelen and J. Norberg of the laboratory staff and the author continued evaluation of the most requested minerals from our collections as potential standards. Although the interest in standards was very broad, our efforts were focused only on silicate materials.

Over a period of approximately 10 years (1968–1978), 31 standards were characterized and made available for distribution [5]. These standards have been widely used by the geochemical community and their acceptance by the users gave us an additional impetus to continue with characterization of other standards. In the early 1980s, four carbonate standards were prepared for a study of corals [6,7]. At the same time a large crystal of Cr-bearing augite became available with approximately 0.8 % of Cr_2_O_3_, a useful standard for the routine analysis of low-concentration chromium in silicates [8]. An important addition to our reference material collection was the donation of fourteen synthetic single-element REE orthophosphates (plus Y and Sc) by Lynn Boatner of the Oak Ridge National Laboratory. These samples were not chemically analyzed, but based on extensive crystallographic data they were determined to be of stochiometric composition [9]. The Corning Glass Company prepared three glasses, each containing 0.75 % of seven elements commonly found in minor quantities in silicate minerals. Paul Carpenter, of the California Institute of Technology, transferred these glasses to our collection in 1997 for distribution. We also recently obtained from the Corning Museum four glass standards used in a study of ancient glasses [10], with a useful range of elements for special applications (Vicenzi et al., see p. 719 of this Special Issue).

## 3. Preparation of Standards

The preparation of standards is a time-consuming and exacting process. Most of the standards used in geological studies are natural minerals, although synthetic materials are also widely used. With both types of materials, the spatial homogeneity was determined by electron microprobe before any wet-chemical analyses were undertaken (Jarosewich et al., 1980). Then, the stability under the electron beam was evaluated. This step requires care, as some samples initially appear to be stable, yet count rates change when the sample is subjected to the electron beam for a prolonged period. For example, dolomite is stable under standard operating conditions (15 kV, 15 μA, 5 μm beam diameter) for about 40 s to 50 s, but after that the count rate changes. For carbonates and high-sodium silicates, most of which are not stable under the electron beam, but are essential standards, special techniques, such as wide beam diameters or rastering are used.

An adequate quantity of standard material should be available for both current and future applications. The quality of material is also important. Ideally, a material of gem quality would make the most desirable standard. However, gem quality samples are difficult to obtain. Frequently mineral separates, consisting of large amounts of small crystals, are used for the preparation of standards because larger single-crystal specimens are not available as in case of South African garnets. Also, mineral separates are more commonly homogeneous than large crystals, yet they require more care in purification than the large crystals due to the presence of impurities and accessory minerals. Usually a Franz magnetic separator, heavy liquids, and/or a microscope is used to separate the impurities and accessory minerals. As a further caution, when selecting minerals for standards it cannot be assumed that all minerals from the same locality are of the same composition. Different fragments must be checked before any work is undertaken. It is important to emphasize that the composition of a given standard is valid only for the characterized sample and other specimens from the same locality may not necessarily be of identical composition.

## 4. Conclusions

The suite of standards characterized by the Smithsonian over many years was an effort outside of normal staff functions; it was done primarily to satisfy the analytical needs of our own staff. Accessibility to minerals from our collection made this task easier. The Smithsonian Microbeam Standards fill only a small part of the need for geological standards. A common practice among users has been to obtain materials from different institutions without regard for proper documentation of the source and composition. Now, with the increasing emphasis on quality control and accreditation of laboratories, there is a growing demand for reliable standards.

Unfortunately, there is only limited institutional support for developing geochemical standards; individuals within various organizations have been doing most of the work. Recently, the United States Geological Survey, together with the newly formed Association of Geoanalysts and other individuals in various institutions have been actively engaged in the characterization of new geochemical standards. Much more needs to be done in preparation of new standards and especially in the timely characterization of these standards by the collaborators.

Standards for trace-element analyses will be increasingly in demand and the materials for such standards will present a considerable challenge in characterization, particularly in establishing homogeneity on the micrometer scale. Aside from the research community, there is a growing demand for major, minor, and trace element microprobe standards of acceptable precision and accuracy from legal and regulatory agencies. The geochemical community must make a concerted effort to meet these requirements.

## Figures and Tables

**Table 1:** List of microbeam standards

Name of sample	Museum number	Reference
Anorthite, Great Sitkin Island, AL	USNM 137041	5
Anorthoclase, Kakanui, New Zealand	USNM 133868	5
Apatite, (Fluor), Durango, Mexico	USNM 104021	1
Augite, Kakanui, New Zealand	USNM 122142	5
Augite, (Cr), Ney County Nevada	NMNH 164905	8
Benitoite, San Benito County, CA	USNM 86539	5
Calcite, Unknown locality	USNM 136321	6
Chromite, Tiebaghi Mine, New Caledonia	USNM 117075	5
Corundum, synthetic	USNM 657S	5
Diopside, Natural Bridge, NY	USNM 117733	5
Dolomite, Oberdorf Austria	USNM 10057	6
Fayalite, Rockport, MA	USNM 85276	5
Gahnite, Brazil	USNM 145883	5
Garnet, Roberts Victor Mine, South Africa	USNM 87375	5
Garnet, Roberts Victor Mine, South Africa	USNM 110752	5
Glass, Basaltic, Juan de Fuca Ridge	USNM 111240 VG-2	5
Glass, Basaltic, Makaopuhi Lava Lake, HI	USNM 113498 A-99	5
Glass, Rhyolitic, Yellowstone Nat. Park, WY	USNM 72854 VG-568	5
Glass, Reference “A” (to be published)	USNM 117218.4	
Glass, Reference “B” (to be published)	USNM 117218.1	
Glass, Reference “C” (to be published)	USNM 117218.2	
Glass, Reference “D” (to be published)	USNM 117218.3	
Glass, IR-V (to be published)	USNM 117083	
Glass, IR-W (to be published)	USNM 117084	
Glass, IR-X (to be published)	USNM 117085	
Glass, Tektite, synthetic	USNM 2213	5
Hornblende, Arenal Volcano, Costa Rica	USNM 111356	5
Hornblende, Kakanui, New Zealand	USNM 143956	5
Hypersthene, Johnstown meteorite	USNM 746	5
Ilmenite, Ilmen Mnts., USSR	USNM 96189	5
Magnetite, Minas Gerais, Brazil	USNM 96189	5
Microcline, location unknown	USNM 143966	5
Olivine, San Carlos, AZ	USNM 111312/44	5
Olivine, Springwater meteorite	USNM 2566	5
Omphacite, Roberts Victor Mine, South Africa	USNM110607	5
Osumilite, Nain, Labrador	USNM 1439667	5
Plagioclase (Labradorite) Lake County, OR	USNM 115900	5
Pyrope, Kakanui, New Zealand	USNM 143968	5
Quartz, Hot Springs, AR	USNM R17701	5
Scapolite (Meionite), Brazil	USNM R6600-1	5
Siderite, Ivigtut, Greenland	USNM R 2460	6
Strontianite, Oberdorf, Austria	NMNH R 10065	7
Rare earth orthophosphates		
CaPO4	USNM 168484	9
DyPO4	USNM 168485	9
ErPO4	USNM 168486	9
EuPO4	USNM 168487	9
GdPO4	USNM 168488	9
HoPO4	USNM 168489	9
LaPO4	USNM 168490	9
LuPO4	USNM 168491	9
NdPO4	USNM 168492	9
PrPO4	USNM 168493	9
SmPO4	USNM 168494	9
ScPO4	USNM 168495	9
TbPO4	USNM 168496	9
TmPO4	USNM 168497	9
YbPO4	USNM 168498	9
YPO4	USNM 168499	9

## 5. Appendix A. List of Microbeam Standards

Compositions of the Smithsonian Microbeam Standards are given in Table 1 of this Appendix. These samples are available upon request by interested researchers.

## References

[1] Mason B (1966). Pyrope, augite, and hornblende from Kakanui, New Zealand. New Zealand J Geol Geophys.

[2] Jarosewich E (1972). Chemical Analysis of Five Minerals for Microprobe Standards. Smithsonian Contrib Earth Sci.

[3] Jarosewich E (1975). Chemical Composition of Two Microprobe Standards. Smithsonian Contrib Earth Sci.

[4] Jarosewich E, Parkes AS, Wiggins LB (1979). Microprobe Analysis of Four Natural Glasses and One Mineral: An Interlaboratory Study of Precision and Accuracy. Smithsonian Contrib Earth Sci.

[5] Jarosewich E, Nelen JA, Norberg JA (1980). Reference Samples for Electron Microprobe Analysis. Geostand Newslett.

[6] Jarosewich E, MacIntyre IG (1983). Carbonate reference samples for electron microprobe and scanning electron microscope analyses. J Sedimentary Petrol.

[7] Jarosewich E, White JS (1987). Strontianite reference samples for electron microprobe and SEM analyses. J Sedimentary Petrol.

[8] Jarosewich E, Gooley R, Husler J (1987). Chromium augite—A new microprobe reference sample. Geostand Newslett.

[9] Jarosewich E, Boatner L (1991). Rare-earth element reference samples for electron microprobe analysis. Geostand Newslett.

[10] Brill RA (1999). Chemical analyses of early glasses.

